# Comment on “An update on toxicology of aluminum phosphide”

**DOI:** 10.1186/2008-2231-20-50

**Published:** 2012-10-08

**Authors:** Omid Mehrpour

**Affiliations:** 1 Birjand Atherosclerosis and Coronary Artery Research Center, Birjand University of Medical Science, Birjand, Iran; 2 Medical Toxicology and Drug Abuse Research Center (MTDRC), Birjand University of Medical Science, Pasdaran Avenue, Birjand, Iran; 3 Medical Toxicology Research Center, Medical School, Mashhad University of Medical Sciences, Mashhad, Iran

## Sir

I read with interest the recent published article by Dr Moghadamnia titled “An update on toxicology of aluminum phosphide” [1]. Since aluminum phosphide (AlP) poisoning is an important medical concern in Iran, I have had the opportunities to work and publish many papers in this regard [2-11]. I would like to comment on that paper as follows:

The author stated that" Leukopenia indicates severe AlP toxicity" and also mentioned that "Increased serum glutamic oxaloacetic transaminase (SGOT) or serum glutamic pyruvic transaminase (SGPT) and induced metabolic acidosis indicate moderate to severe AlP overdose". Leukopenia and hepatotoxicity due to AlP poisoning is reported in the literature, but according to my knowledge there is no study supported that they are good indicators of severity of AlP poisoning [2,3]. Even some studies indicated that leukosytosis is known as a prognostic factor in AlP poisoning [3]. Shadnia et al. in a study found that the prognostic factors in AlP poisoning are Simplified Acute Physiology Score II (SAPSII), low GCS, hypotension, hyperglycemia, acidosis, hemoconcentration, leukocytosis, hyperuremia and ECG abnormalities [3].

Moreover the author stated that" The serum level of cortisol is usually decreased in severe AlP poisoning". Very few studies have published about blood cortisol level changes in AlP poisoning. Generally in clinical situations such as stress, hypotension, shock or critically ill patients, there is evidence of increasing in plasma cortisol level. In addition, some surveys showed that AlP poisoning has been postulated to stimulate cortisol, glucagon, and adrenaline secretion, or inhibit insulin synthesis [6,12]. Moreover Chugh, et al. (1989) reported findings in thirty cases that showed a significant rise in plasma cortisol (greater than 1048 nmol/L) in 20 cases [13]. In our previous study we found that Twenty-three (71.9%) of non-survived and four (30.8%) of survived patients of AlP poisoning had a blood glucose level greater than 140 mg/deal and suggested the possibility of increasing in plasma cortisol level in AlP poisoning [6]. I will encourage the toxicologists to provide studies for evaluating the plasma cortisol level in AlP poisoning in the future.

In addition the author accentuated the role of charcoal in AlP poisoning. I agree that administration of charcoal in this fatal poisoning may be useful and in fact charcoal administration is better than its missing. Although charcoal is generally used for decreasing phosphine absorption in the gastrointestinal system, no study has demonstrated its efficacy in humans [2,14]. We encourage to provide studies for evaluating its efficacy for absorption of phosphine in vitro and in vivo.

## Competing interests

The author declare that he has no competing interests.
